# The Influence of an Anti-Inflammatory Gluten-Free Diet with EPA and DHA on the Involvement of Maresin and Resolvins in Hashimoto’s Disease

**DOI:** 10.3390/ijms252111692

**Published:** 2024-10-30

**Authors:** Małgorzata Szczuko, Julia Kacprzak, Aleksandra Przybylska, Urszula Szczuko, Jakub Pobłocki, Anhelli Syrenicz, Arleta Drozd

**Affiliations:** 1 Department of Human Nutrition and Metabolomics, Pomeranian Medical University, 71-460 Szczecin, Polandola.przybylska99@gmail.com (A.P.); arleta.drozd@pum.edu.pl (A.D.); 2 Department of Human Nutrition and Bromatology, Pomeranian Medical University, 71-210 Szczecin, Poland; 3 Department of Endocrinology, Metabolic Diseases and Internal Diseases, Pomeranian Medical University, 70-252 Szczecin, Poland; jakub.poblocki@pum.edu.pl (J.P.); anhelli.syrenicz@pum.edu.pl (A.S.)

**Keywords:** Hashimoto’s thyroiditis, gluten-free diet, fatty acid derivatives, inflammatory state, EPA, DHA, resolvin, protectin, maresin

## Abstract

The potential modulation of thyroid inflammatory conditions via a gluten-free diet has been suggested after establishing a link between Hashimoto’s thyroiditis (HT) and celiac disease. However, the majority of targeted studies in this field do not support the general recommendation of prescribing a gluten-free diet (GFD) for all HT patients. This study aims to analyze data regarding the impact of a GFD supplemented with eicosapentaenoic (EPA) and docosahexaenoic acid (DHA), along with vegetables, on the course of inflammation involving long-chain fatty acid mediators. The study cohort consisted of 39 Caucasian female patients with autoimmune HT. Metabolite separations were performed using a liquid chromatograph with a DAD detector. Absorption peaks were read at 210 nm for resolvin E1, protectin DX, and maresin 1 and at 302 nm for resolvin D1. The introduction of a gluten-free diet completed with omega-3, including EPA and DHA, may contribute to a reduction in the inflammatory state in HT patients. This effect is supported by the elevation in the levels of anti-inflammatory mediators derived from long-chain fatty acids with anti-inflammatory properties but not by eliminating gluten. Significant statistical changes in the levels of all derivatives were observed before and after the implementation of the diet. It is worth noting that this effect was not observed in anti-TPO and anti-TG levels. The induction of anti-inflammatory changes can be achieved by supplementing the diet with EPA, DHA and vegetables with increased anti-inflammatory potential.

## 1. Introduction

The clear predominance of Hashimoto’s disease incidence among women confirms the sexual dimorphism of the immune system. Women have a higher number of CD4+ lymphocytes, an increased ratio of CD4+ to CD8+ subpopulations and a preference for a Th2-dependent response. The disease particularly affects white women, which indicates a genetic basis of the disease [1]. The therapy for Hashimoto’s disease involves a substitutive treatment of thyroid insufficiency, achieved by replenishing the lacking hormones initially produced in the continuously targeted thyroid gland [2]. To counteract the progressive destructive process, anti-inflammatory measures must be introduced (anti-inflammatory diet, regular physical activity, supplementation, stress reduction, and decreased toxin exposure). Moreover, while suffering from one autoimmune-based disorder, the susceptibility to other autoimmune diseases is higher. As chronic Hashimoto’s thyroiditis (HT) progresses, the inflammation increases, initiating the onset of various conditions, such as gluten intolerance, joint inflammation, or dermatological issues [2]. Consequently, patients for whom hormone therapy fails to alleviate the symptoms usually expand their treatment to incorporate steroidal or non-steroidal anti-inflammatory drugs. Steroids cause numerous side-effects, thus prompting the exploration of more natural substances with the potential to reduce inflammatory states and yield tangible outcomes [1,2].

Through the association of HT with celiac disease (CD), a GFD has been suggested to potentially modulate thyroid inflammatory conditions [3]. However, in studies where patients with and without celiac disease were involved, the reduction in gluten intake led to diminished thyroid inflammation solely in individuals afflicted with CD, with no significant impact observed on thyroid peroxidase antibodies (anti-TPO). Furthermore, patients with celiac disease are more susceptible to autoimmune thyroid diseases [3]. Currently, the degree of evidence supporting the recommendation of a gluten-free diet for thyroid inflammation patients without celiac disease remains insufficient [4] despite its proposition as a therapeutic strategy in autoimmune diseases unrelated to celiac disease [5]. This cautious approach stems from a recent review article addressing potential nutritional deficiencies observed in patients adhering to a strict gluten-free diet [6]. Therefore, individuals with Hashimoto’s thyroiditis should undergo a screening for transglutaminase antibodies (anti-TG), deamidated gliadin peptide antibodies (anti-DPG), endomysium antibodies (EMA), and anti-gliadin antibodies (AGA) to rule out subclinical CD and non-celiac gluten sensitivity (NCGS) [7]. There is increasing interest in the use of a gluten-free diet (GFD) in patients with Hashimoto’s disease to alleviate its symptoms. Research indicates a connection between this endocrinological disorder and celiac disease, which is directly related to the reaction to gluten. In a significant proportion of individuals with celiac disease (around 10%), anti-thyroid antibodies are detected. Conversely, patients with Hashimoto’s disease are more likely to have positive results for celiac markers, specifically, IgA antibodies to tissue transglutaminase. In addition, a correlation between TSH levels and IgG antibodies against gliadin has been observed [8,9].

HT may be part of polyglandular autoimmune syndrome type 2 (PAS-2) along with autoimmune adrenal insufficiency (Addison’s disease) and type 1 diabetes, known as Carpenter’s syndrome [10]. Inflammatory thyroid diseases (AITDs) are also clearly associated to “non-thyroid” autoimmune diseases (NTADs), such as adrenal insufficiency, pernicious anemia and CD. As a result, patients experience a broad spectrum of symptoms affecting both their physical and psychological well-being [11]. The etiology of the disease highlights the importance of genetic predispositions in Hashimoto’s development [12]. However, environmental factors are essential to trigger the immune attack and thus the inflammatory state [13]. Stress, infections [14], microbiota dysbiosis [15], and malnutrition, including qualitative deficiencies (vitamin D and cobalamin), are recognized as key contributors [16].

It has been demonstrated that omega-3 fatty acids, eicosapentaenoic acid (EPA), and docosahexaenoic acid (DHA) alleviate inflammatory processes. However, the molecular mechanism of this process is not entirely clear. Potential molecular factors contributing to the anti-inflammatory properties of omega-3 PUFAs include the G-protein-coupled receptor 120 (GPR120) and the peroxisome proliferator-activated receptor γ (PPARγ) [17]. The conversion of omega-3 fatty acids into anti-inflammatory resolvins and protectins, as well as the identification of putative target GPCRs (G-protein-coupled receptors), such as ChemR23, BLT1, ALX/FPR2, and GPR32, are of great interest for reduction in inflammatory states [18].

Identifying the impact of omega-3 fatty acids on other key molecular factors, such as leukotrienes and prostaglandins and their signals may aid in recognizing and developing drugs that inhibit major pro-inflammatory mediators and induce the expression of anti-inflammatory cytokines and nuclear receptors. Bioactive lipid mediators play a role in signaling, particularly in the process of neutralizing inflammatory states. EPA and DHA can modulate the nuclear factor κB (Nf-κB) activation pathway [19]. Until now, it was assumed that the resolution of the inflammatory response is a slow process based on the suppression of pro-inflammatory signaling pathways. It is a highly complex process controlled via various mediators that leads to restoring homeostasis. It can persist long after the acute phase of injury has ended. Without resolution, the body never truly returns to homeostasis, leading to chronic inflammation [20].

Specialized pro-resolving mediators (SPMs) represent a portion of the spectrum of omega-3 fatty acid derivatives, which have a significant impact on reducing inflammation [20]. They can effectively resolve inflammatory states without weakening the immune response, and they do not exhibit risky side-effects [21]. These mediators could become potential therapeutic targets for inflammatory diseases [22]. Researchers are currently working on understanding how these mediators impact the inflammation progress [23].

Studies show that omega-3 fatty acid derivatives, such as resolvins (Rv), maresins (MaR), and protectins (PD), have strong anti-inflammatory effects in the progression of many diseases (arthritis, cancer, and atherosclerosis) associated with inflammation, such as Hashimoto’s disease. New substances with anti-inflammatory potential and safer effects than currently used standard steroidal and non-steroidal anti-inflammatory drugs are being investigated, hence the interest in the potential of omega-3 fatty acid derivatives [24]. Maresin-1 demonstrates versatile effects on the immune system, affecting dendritic cells (DCs)/macrophages, regulatory T cells and neutrophils [25]. In addition to its anti-inflammatory action, maresin suppresses neutrophil migration and cytokine production by activating CD8+ T cells, CD4+ T helper (Th1) cells, and Th17 cells [26]. It appears that maresin negatively regulates the transcription of factors T-bet and Rorc, thereby preventing the differentiation of Th1 and Th17 cells and simultaneously enhancing the generation of Foxp3+ regulatory T cells (Tregs) via the GPR32 receptor [20]. The changes in the levels of all anti-inflammatory derivatives showed statistically significant differences before and after dietary intervention.

Two receptors involved in the mechanism of action of maresin-1 have been discovered. One is the leucine-rich G-protein-coupled receptor 6 (LGR6), which has a GPCR-like structure and is identified in various tissues [27]. The second receptor is the retinoic acid-related orphan receptor alpha (RORα), located in the nucleus [28]. However, the exact molecular mechanism of inflammation resolution, systemic defense, or cellular homeostasis has not been described. Therefore, it is not possible to accurately predict the impact of maresin on inflammation reduction.

In our study, we implemented a GFD completed with EPA and DHA alongside vegetables possessing anti-inflammatory properties. The authors expected the anti-inflammatory effect of EPA and DHA acids. However, they wanted to explain why there is so much contradiction and controversy surrounding this diet in the treatment of Hashimoto.

Similarly, the impact of related cytokines in the pathogenesis pathway, cross-reacting antibodies, or malnutrition due to deficiencies in various essential micronutrients for proper thyroid function, especially iron and vitamin D, also plays a role [29]. An appropriate diet and adherence to its regimen could significantly improve thyroid function and reduce autoantibody reactivity in Hashimoto’s thyroiditis [30].

Our aim was to analyze and present the knowledge regarding the impact of a GFD supplemented with EPA and DHA, along with vegetables, on the course of inflammation involving long-chain FFA (free fatty acids) anti-inflammatory mediators in Hashimoto’s disease. The study assumed that eliminating gluten from the diet and supplementing long-chain fatty acids may support the treatment of thyroid inflammation. However, the involvement of pathways synthesizing omega-3 derivatives in reducing inflammation by enhancing the synthesis of resolvins, protectins and maresins will not be evident. Replacing gluten-containing grains with vegetables and products containing EPA and DHA may have anti-inflammatory potential not directly related to the elimination of gluten. Checking the effect of substances with anti-inflammatory potential in Hashimoto’s disease seems to be the next stage of research.

## 2. Results

The biochemical parameters of the patients who participated in this study were evaluated, including anti-TPO antibodies, anti-TG antibodies and the following hormones: TSH, fT3, fT4, and CRP. The average results and SD are presented below in the table. The mean values were within the reference range except for anti-TPO and anti-TG antibodies (Table 1).

### 2.1. Comparison of the Mediator Levels Before and After the Implementation of the GF Diet

**Figure 1:**
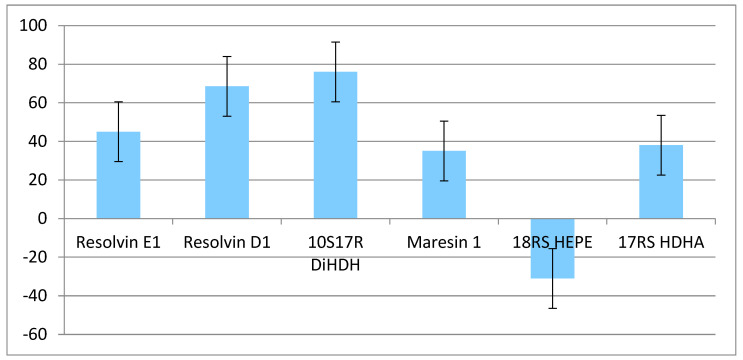
Difference in the change in fatty acid derivatives level [%]. 10(S)17(R)-DiHDAs protectin DX.

When comparing the values of anti-inflammatory mediators before and after the diet, an increase was observed in almost all of them (Figure 1). The level of resolvin E1 increased by 45%, resolvin D1 by 68.5%, 10S17R DiHDHA by 76%, maresin 1 by 35%, and 17RS HDHA by 38%. Only the level of 18RS HEPE decreased by 31% (Figure 1). As a result of this study, a significant increase in the level of resolvin D1, as well as resolvin E1, 10S17R DiHDHA, 17RS HDHA, and maresin 1, was observed in the study group after the diet. A decrease was observed for 18RS HEPE (Figure 1). The changes in the levels of all derivatives were statistically significant (Table 2).

### 2.2. Correlation of the Fatty Acid Derivatives with the Anthropometric Parameters Before and After the Implementation of the Diet

As a result of the correlation of anti-inflammatory derivatives with anthropometric measurements, it was found that statistically significant values for both resolvins (D1 and E1) were found concerning the fat tissue mass in [%] and [kg] before the implementation of the diet. The body weight of patients and BMI were statistically significantly correlated with resolvin D1 (Table 3). The test showed that these were positive correlations of average strength. On the other hand, 17RS HDHA was significantly correlated with height (Table 3).

When examining the correlations of fatty acid derivatives after the diet, statistically significant correlations were observed only for age and 18RS HEPE, as well as resolvin E1 and height (Table 4).

### 2.3. Correlations of Fatty Acid Derivatives with Biochemical Parameters Before and After the Implementation of the Diet

When analyzing the correlations of mediators with biochemical parameters, several statistically significant patterns were observed before the implementation of the diet (Table 5).

There were significant correlations of fT4 with maresin 1 and 17RS HDHA. The latter also significantly correlated with TG antibodies. On the other hand, 10S17R DiHDHA significantly correlated with the level of TSH. After the diet, only one correlation was found, the one between 18RS HEPE and fT3 (Table 6)

In summary, as a result of the study of the correlation of the level of inflammatory mediators with the anthropometric parameters of patients, a significant correlation of resolvin E1 and resolvin D1 with the percentage content of adipose tissue and the mass of adipose tissue (expressed in kg) before the diet was demonstrated. Regarding biochemical parameters, statistical significance was demonstrated for the correlation of maresin 1 and 17RS HDHA with fT4, 10S17R DiHDHA with TSH, and 17RS HDHA with anti-TG before the diet. On the other hand, 18RS HEPE was statistically significantly correlated with the level of fT3 after the diet. Resolvin D1 may have the greatest potential to positively influence inflammation in inflammatory thyroid disease, Hashimoto’s thyroiditis, as it lowers both anti-TPO and anti-TG antibody levels. It stimulates thyroid function by increasing the conversion of fT4 to fT3. Maresin 1 was statistically significantly correlated with fT4 levels. This correlation was positive with average strength. 18RS HEPE was significantly associated with the level of fT3. This correlation was negative and showed average strength. 17RS HDHA was significantly associated with fT4 and Anti-TG. This correlation was positive and of average strength.

## 3. Materials and Methods

### 3.1. Characteristics of the Study Group

The study group consisted of 39 Caucasian female patients with autoimmune Hashimoto’s thyroiditis (HT), with an average age of 37.393 ± 8.097. The anthropometric parameters of the women were measured (height, body weight, BMI, and fat tissue mass). Almost all patients were characterized by a reduced activity level at the level of activity coefficient 1.3–1.4. The patients were newly diagnosed within two years. The most frequently reported clinical symptoms of Hashimoto’s disease were fatigue, weight gain, feeling of cold limbs, dry skin, enlargement of the thyroid gland in the ultrasound and swelling in the front of the neck, with muscle pain. Less frequently reported were hair loss, sleep and concentration disorders, difficulty swallowing, joint pain, and mood disorders, including depression and anxiety.

The biochemical parameters related to thyroid function were also examined and compared, including anti-TPO antibodies, anti-TG antibodies, thyroid-stimulating hormone (TSH), triiodothyronine (fT3), thyroxine (fT4), and C-reactive protein (CRP). HT was diagnosed on the basis of a typical ultrasound image of chronic autoimmune thyroiditis (using an ALOKA Prosound Alpha-7 device with a UST-5411 4.4–13.3 MHz, linear probe Japan) and elevated antibody levels in blood serum. The exclusion criteria included the prior use of a gluten-free diet or periodic gluten elimination, malabsorption syndromes, bariatric surgery, intestine or stomach resection, thyroidectomy, Graves’ disease or its clinical symptoms, diabetes, hypertension, coronary artery disease and use of gluco-corticosteroids, non-steroidal anti-inflammatory drugs, immunosuppressants, or drugs affecting the thyroid axis other than levothyroxine. Due to hypothyroidism, 37 patients received levothyroxine replacement therapy and were clinically euthyroid. The patients were thoroughly informed of the study and provided informed consent. All patients gave written consent to participate in the study. The description of the study group based on anthropometric data is presented in Table 7. After the diet, body weight and BMI parameters decreased insignificantly and were respectively 69.97 ± 10.98 and 25.07 ± 3.56.

### 3.2. Dietary Intervention

GFD was defined as the consumption of gluten-free natural and processed products containing ≤20 mg gluten per kg. GFD compliance was verified by a clinical dietitian. All participants received a sample GFD menu supplemented with EPA and DHA (fish, algae, seafood). All participants received a sample GFD menu in the range of 1800–2000 kcal. Follow-up visits with a clinical dietitian included analysis of patients’ food diaries and products containing EPA and DHA or vegetable oil precursors (linseed oil, avocado, nuts). Follow-up visits took place every two months. The diet was followed for a minimum of 6 months. The estimated daily intake of omega-3 fatty acids from the menu was 4–7 g per day. Adherence to the GFD was analyzed using an adherence questionnaire [31]. Only patients with excellent (n = 10), good (n = 23) and satisfactory (n = 6) adherence according to the questionnaire were included in the GFDG study [31].

### 3.3. Eicosanoid Extraction

Eicosanoids were extracted from blood serum using RP-18 solid-phase extraction columns (Agilent Technologies, Cheadle, UK) for solid-phase extraction: 10(S)17(R)-DiHDAs (protectin DX), maresin, resolvin D1, resolvin E1. For eicosanoid extraction, 0.5 mL of serum was added to 1 mL of acetonitrile for protein precipitation, and 50 µL of internal standard (1 µg/mL) was added. After incubation for 15 min at −20 °C, the samples were centrifuged for 10 min using a cooling centrifuge (Eppendorf, centrifuge 5804R). The pH of each sample was adjusted to 3 by adding 30–50 µL of 1 M HCl. The columns were activated by successively washing with 3 mL of 100% acetonitrile and 3 mL of 20% acetonitrile in water. The samples were loaded and double-washed with 20% acetonitrile in water. Eicosanoids were subsequently eluted with a mixture of methanol and ethyl acetate (1:1 *v*/*v*), dried under vacuum, and dissolved in 60% methanol in water with 0.1% acetic acid [32,33].

### 3.4. HPLC Operating Parameters

HPLC separations were performed using an Agilent Technologies 1260 liquid chromatograph with Agilent ChemStation software (B.0404; Agilent Technologies, Cheadle, UK). The separation was conducted using a Thermo Scientific Hypersil BDS C18 column (100 × 4.6 mm, 2.4 µm) (Agilent Technologies, Cheadle, UK). The column oven temperature was set to 20 °C. The content of buffer B in the mobile phase was 30% from 0 to 2 min of separation, linearly increased to 80% at 33 min, held at 98% between 33.1 and 37.5 min, and then reduced to 30% between 40.3 and 45 min. The flow rate was 1.0 mL/min. The sample injection volume was 60 µL. A DAD detector monitored peaks by absorption at 210 nm for resolvin E1, protectin PD (10S17R_DiHDHA), maresin-1, and at 302 nm for 5(S), 6(R), 15(R)-resolvin D1. The absorbance spectra of peaks were analyzed to confirm analyte identification [33].

### 3.5. Statistical Analysis

Statistical analyses were performed using MedCalc^®^ Statistical Software (version 20.218; MedCalc Software Ltd., Ostend, Belgium; https://www.medcalc.org). Parametric tests were used because the distribution was mostly normal (Shapiro–Wilk test). The paired samples *t*-test was used to compare the means of two measurements taken on the same patient. Correlations between fatty acid derivative levels and biochemical test results were analyzed using Pearson’s test (Pearson correlation coefficient). Linear relationships (r) between two quantitative variables were depicted as positive within the range of 0.3 to 1 and negative within the range of −0.3 to −1.

## 4. Discussion

Knowledge of how lipids mediators are regulated during different inflammatory responses is limited and requires further investigation. Recent findings [34] have shown that traditional anti-inflammatory drugs such as celecoxib and microRNA molecules were effectively delivered by SPMs and altered the inflammatory profile of naive (M0φ) and M1-primed (M1φ) macrophages as assessed by gene and protein expression. These findings suggest a good prognosis for modulating the initial immune response by reducing the risk of chronic inflammation [35]. SPMs, such as protectins, maresins and D-series resolvins, act as biased positive allosteric modulators (PAMs) of the prostaglandin E 2 (PGE 2) EP4 receptor via an intracellular binding site. They endow the endogenous EP4 receptor on macrophages with the ability to bind to G-proteins, thereby converting the EP4 receptor on macrophages from an antiphagocytic receptor to a receptor that enhances phagocytosis, a central mechanism of pro-inflammatory activity [36].

Analyzing the derivatives of long-chain omega-3 fatty acids in our study, it was observed that resolvins, especially the derivatives of DHA, have a favorable impact on the levels of bio-chemical parameters associated with Hashimoto’s disease. Both analyzed resolvins, RvE1 and RvD1, were associated with a decrease in the level CRP and anti-TG levels, as well as fT4 levels, while increasing fT3 levels. However, only RvD1 affected reductions in anti-TPO levels, antibodies directed against thyroid-specific antigens. In summary, the results obtained suggest that a diet rich in omega-3 fatty acids, mainly DHA, the source of RvD1, has a beneficial effect on the course of HT.

Diagnostic tests for gluten sensitivity/intolerance in HT patients are necessary as the risk of celiac disease is several times higher in their case [37,38,39]. By treating celiac disease with a gluten-free diet, we can simultaneously reduce overall inflammation, including thyroid-related inflammation, thus alleviating symptoms of thyroid inflammatory disease [40]. A gluten-free diet supplemented with vegetable oils rich in omega-3 fatty acids, EPA and DHA may have anti-inflammatory potential at the thyroid level. However, the observed effect should be considered an adjunctive result of the gluten-free diet incorporating other modifications (partial replacement of gluten products with increased vegetable consumption). In this study, patients supplemented their diet with additional omega-3 fatty acids, so for now, we can recommend a balanced diet rich in EPA and DHA sources (primarily fish and their products, algae and seafood). Research examining the impact of a gluten-free diet on thyroid-related parameters showed no differences in TSH, fT3, and fT4 concentrations in the study group after the gluten-free diet [41]. Anti-TPO and anti-TG antibody levels were reduced, and 25(OH)D levels slightly increased [13,41]. Diverse results from analyzed dietary interventions could stem from varying thyroid conditions, substantial variability among patients, and differences in customary intake of key nutrients (such as iodine, selenium and iron) across different populations [13]. In the study by Velija et al., a larger number of patients receiving selenium supplementation whilst adhering to a gluten-free diet restored euthyroidism at six months compared to the control group receiving selenium supplementation without dietary intervention. Additionally, the decrease in serum anti-TPO antibody levels was significantly greater in the study group than in the control group. These findings suggest that a gluten-free diet combined with selenium supplementation is more effective than selenium supplementation alone in women with HT and subclinical hypothyroidism [42]. Currently, there is not enough evidence to recommend this diet to support therapy for autoimmune diseases other than celiac disease [4,7].

It has been shown that omega-3 derivative compounds enhance the synthesis of resolvins, particularly RvD1 [43]. In this study, after implementing a gluten-free diet supplemented with omega-3 fatty acids, the levels of 10S17R DiHDHA, RvD1, RvE1, 17RS HDHA, and maresin 1 increased significantly, while only the level of 18RS HEPE decreased. Statistically significant differences were observed in the levels of all anti-inflammatory mediators before and after the dietary intervention.

Anti-TPO is the most sensitive parameter in detecting autoimmune thyroid diseases, as TPO (thyroid peroxidase) is the primary antigen of thyroid cell microsomes. Consequently, the level of anti-TPO is crucial in monitoring the level of inflammation in inflammatory thyroid disease. It has been observed that only RvD1 can reduce the level of these antibodies. It can also affect CRP, a key indicator of systemic inflammation, anti-TG, and fT4, while increasing TSH and fT3, indicating thyroid stimulation and enhanced conversion of fT4 to fT3. This highlights the significance of RvD D1, a derivative of DHA (docosahexaenoic acid) from the long-chain omega-3 fatty acid family, in the inflammatory process of thyroid disease. The serum level of RvE1 in HT patients was significantly lower than in healthy subjects [44]. Reduced RvE1 levels could indicate that HT is linked to the impaired resolution of inflammation. RvE1 may serve as a protective factor against elevated TgAb levels [44].

A similar study measuring serum resolvin D1 (RvD1) levels in the serum of patients with Hashimoto’s thyroiditis (HT) and healthy individuals in the control group, along with an analysis of the correlation between RvD1 and thyroid autoantibodies and inflammatory factors, demonstrated a negative correlation between serum RvD1 levels and anti-TPO levels in paired samples. This implies that as anti-TPO levels increase, RvD1 level tends to decrease, suggesting that RvD1 is utilized. In another study RvD1 exhibited a negative correlation with the inflammatory chemokine IP-1, which participates in the inflammatory process by stimulating the migration of leukocytes and other cells to inflammatory sites [45]. Thyroid autoimmunity is therefore associated with decreased RvD1 levels. However, it is not known whether RvD1 is associated with other autoimmune conditions. Reduced RvD1 levels contribute to impaired resolution inflammation in HT patients and the results of this study indicate that adopting the described dietary approach can significantly increase RvD1 levels. Furthermore, it was found that RvD1 negatively correlates with Anti-TPO, implying that increasing RvD1 levels induced by the diet could potentially reduce inflammation and antibody levels. Nevertheless, the final statement requires further research.

In another study examining the effects of DHA (docosahexaenoic acid) on liver inflammation, it was found that simultaneous administration of DHA and fT3 (thyroid hormone) enhances the liver’s ability to resolve inflammation by increasing the availability of RvD1. This is an important mechanism of liver protection in clinical conditions [46]. The results described in this study demonstrate that RvD1 influences the reduction in CRP, anti-TPO and anti-TG levels while increasing fT3 levels. Based on the referenced study, it can be inferred that RvD1 not only reduces thyroid inflammation but also aids in resolving liver inflammation and the conversion of fT4 to its active hormone form.

The results obtained in this study indicate that in combination with omega-3 fatty acid supplementation, a gluten-free diet positively impacts the course of thyroid inflammation, which was the result of supplementation with long-chain fatty acids rather than just gluten elimination. It decreases the severity of inflammation by increasing the levels of long-chain fatty acid derivatives (resolvins E1 and D1, maresin 1, 17RS HDHA, and 18RS HEPE) with antioxidant properties. Consequently, it has a positive effect on disease progression and can lead to the alleviation of its symptoms. When recommending an appropriate dietary approach for patients with thyroid inflammation, the focus should be on providing adequate omega-3 intake and a balanced diet that meets individual macro- and micronutrient needs [47,48]. Chronic inflammatory diseases such as Hashimoto’s thyroiditis need adequate protein, fat, and carbohydrate intake, as well as vitamins and minerals essential to maintaining the body’s health in its ongoing battle against inflammation. Gastrointestinal health is critical for absorption of thyroid specific nutrients and for direct thyroid function and hormone conversion [49]. Any diets that pose a risk of nutrient deficiency in HT patients would not be suitable in this case without additional supplementation. Therefore, in HT, applying a well-rounded, balanced diet supplemented with LCPUFAs that accelerate the process of reducing chronic inflammation and restoring tissue homeostasis holds greater significance [50,51,52]. In addition, supplementation with fiber and fresh raw vegetables reduces the risk of intestinal dysbiosis and intestinal leakage. Recently, HT patients were found to have elevated levels of zonulin, suggesting leaky gut in these patients [13,53]. A healthy intestinal microflora has a beneficial effect not only on the functioning of the immune system, but also on the functioning of the thyroid gland [14].

A limitation of the study is the relatively small group of Hashimoto’s patients. The group was not homogeneous because two people did not take hormone therapy, but all were euthyroid. All qualified patients were women. While the inclusion of a group of men would be justified, it would be challenging to recruit due to the rarity of the disease. The inclusion of a control group of healthy subjects would be desirable, but difficult to implement over a period of 6 months of dieting.

## 5. Conclusions

The results of this study can play an important role in raising public health awareness of the anti-inflammatory effects of the diet in Hashimoto’s patients and serve as a recommendation for widespread testing of HT patients for gluten intolerance. Based on the results of this study, it was concluded that the implementation of a gluten-free diet in HT patients does not lead to a reduction in antibody levels. The impact of this diet containing EPA and DHA manifests as the potential to reduce inflammation via the involvement of anti-inflammatory derivatives such as 17RS HDHA, 18RS HEPE, maresin 1, resolvin D1, and resolvin E1. This effect is demonstrated by an increase in the levels of anti-inflammatory mediators. However, this is not a result of GFD on the changes in levels of anti-TPO and anti-TG. In fact, the dietary approach may play a significant role in the pathogenesis of HT.

## Figures and Tables

**Table 1:** The characteristics of the study group based on the average biochemical parameters.

Biochemical Parameters	Mean Value ± SD
Anti-TPO [0–34 IU/mL]	180.65 ± 137.51
Anti-TG [0–115 IU/mL]	324.74 ± 540.28
TSH [0.270–4.200 IU/mL]	3.17 ± 2.679
fT3 [2.00–4.40 pg/mL]	2.92 ± 0.467
fT4 [0.93–1.70 ng/dL]	1.25 ± 0.204
CRP [0.00–5.0 mg/L]	2.2 ± 1.406

Anti-TPO—thyroid peroxidase antibodies; Anti-TG—for transglutaminase antibodies; TSH—thyroid-stimulating hormone; fT3—triiodothyronine; fT4—thyroxine; CRP—C-reactive protein.

**Table 2:** Comparison of analyzed metabolites before and after implementation of the diet.

Parameters [μg/mL]	BEFORE		AFTER		*p* Value
	Mean	SD	Mean	SD	
10S17R_DiHDHA	1.1357	2.6205	2.0022	2.6427	<0.001
17RS HDHA	3.3595	1.8931	4.6311	2.4927	<0.003
18RS HEPE	0.3499	0.2667	0.241	0.1815	<0.001
Maresin 1	0.2433	0.1081	0.328	0.1633	<0.001
Resolvin D1	0.1559	0.07717	0.2632	0.1544	<0.004
Resolvin E1	0.7271	0.5141	1.0605	0.6925	<0.002

SD—standard deviation; *p*-value statistically significant when *p* < 0.05; 10(S)17(R)-DiHDAs protectin DX.

**Table 3:** Correlation of fatty acid derivatives with anthropometric parameters of patients before the implementation of the diet.

[µg\mL]	Body Fat Mass [%]	Body Fat Mass [g]	BMI	Body Weight	Height	Age
Resolvin E1	**r = 0.387**	**r = 0.344**	r = 0.297	r = 0.289	r = 0.052	r = −0.109
	***p* = 0.015**	***p* = 0.0319**	*p* = 0.0668	*p* = 0.0743	*p* = 0.7541	*p* = 0.5089
Resolvin D1	**r = 0.386**	**r = 0.356**	**r = 0.328**	**r = 0.339**	r = 0.113	r = −0.064
	***p* = 0.0151**	***p* = 0.0263**	***p* = 0.0413**	***p* = 0.0346**	*p* = 0.4945	*p* = 0.6992
10S17R DiHDHA	r = −0.027	r = −0.116	r = 0.035	r = −0.033	r = −0.177	r = −0.03
	*p* = 0.8707	*p* = 0.4805	*p* = 0.8329	*p* = 0.8413	*p* = 0.2806	*p* = 0.8553
Maresin 1	r = 0.25	r = 0.194	r = 0.001	r = 0.101	r = 0.272	r = 0.112
	*p* = 0.1252	*p* = 0.2362	*p* = 0.9944	*p* = 0.5404	*p* = 0.0941	*p* = 0.4986
17RS HDHA	r = 0.2	r = 0.18	r = −0.031	r = 0.128	r = 0.41	r = 0.043
	*p* = 0.2233	*p* = 0.272	*p* = 0.8532	*p* = 0.4357	***p* = 0.0096**	*p* = 0.7958
18RS HEPE	r = 0.141	r = 0.081	r = 0.028	r = 0.063	r = 0.081	r = −0.096
	*p* = 0.3911	*p* = 0.6229	*p* = 0.8666	*p* = 0.7047	*p* = 0.625	*p* = 0.5612

Bold font indicates statistically significant correlation; 10(S)17(R)-DiHDAs protectin.

**Table 4:** Correlation of fatty acid derivatives with anthropometric parameters of patients after the implementation of the diet.

[µg\mL]	Body Fat Mass [%]	Body Fat Mass [g]	BMI	Body Weight	Height	Age
Resolvin E1	r = 0.118	r = −0.006	r = 0.07	r = −0.018	r = −0.22	r = −0.131
	*p* = 0.4746	*p* = 0.9699	*p* = 0.6706	*p* = 0.9145	*p* = 0.1799	*p* = 0.4257
Resolvin D1	r = 0.142	r = 0.029	r = 0.099	r = −0.05	r = −0.367	r = 0.033
	*p* = 0.3884	*p* = 0.8602	*p* = 0.549	*p* = 0.761	***p* = 0.0214**	*p* = 0.8397
10S17R DiHDHA	r = 0.133	r = 0.184	r = 0.127	r = 0.218	r = 0.286	r = −0.119
	*p* = 0.4182	*p* = 0.2632	*p* = 0.4401	*p* = 0.1822	*p* = 0.077	*p* = 0.4697
Maresin 1	r = −0.085	r = −0.063	r = 0.053	r = −0.054	r = −0.306	r = −0.135
	*p* = 0.6073	*p* = 0.7025	*p* = 0.7477	*p* = 0.743	*p* = 0.058	*p* = 0.4129
17RS HDHA	r = −0.046	r = 0.006	r = −0.134	r = 0.105	r = −0.065	r = 0.073
	*p* = 0.7822	*p* = 0.9702	*p* = 0.4169	*p* = 0.5262	*p* = 0.6922	*p* = 0.6599
18RS HEPE	r = −0.033	r = 0.021	r = 0.182	r = 0.148	r = −0.068	r = 0.326
	*p* = 0.8434	*p* = 0.901	*p* = 0.2673	*p* = 0.3681	*p* = 0.6817	***p* = 0.0428**

Bold font indicates statistically significant correlation.; 10(S)17(R)-DiHDAs protectin.

**Table 5:** Correlation of anti-inflammatory metabolites with biochemical parameters of patients before the implementation of the diet.

[µg\mL]	TSH	Anti-TG	Anti-TPO	fT3	fT4	CRP
Resolvin E1	r = −0.2	r = −0.007	r = 0.048	r = 0.067	r = 0.167	r = 0.253
	*p* = 0.2229	*p* = 0.964	*p* = 0.7726	*p* = 0.6866	*p* = 0.0269	*p* = 0.1202
Resolvin D1	r = −0.016	r = 0.067	r = −0.052	r = 0.066	r = 0.108	r = 0.276
	*p* = 0.9206	*p* = 0.6849	*p* = 0.7546	*p* = 0.6888	*p* = 0.0269	*p* = 0.089
10S17R DiHDHA	r = 0.354	r = 0.016	r = 0.167	r = 0.154	r = −0.163	r = −0.123
	*p* = 0.0269	*p* = 0.9228	*p* = 0.3087	*p* = 0.3488	*p* = 0.0269	*p* = 0.4556
Maresin 1	r = −0.19	r = 0.188	r = −0.151	r = 0.099	**r = 0.317**	r = −0.052
	*p* = 0.246	*p* = 0.2516	*p* = 0.3587	*p* = 0.5475	***p* = 0.049**	*p* = 0.754
17RS HDHA	r = −0.308	r = 0.357	r = −0.177	r = 0.119	**r = 0.333**	r = 0.101
	*p* = 0.566	***p* = 0.0256**	*p* = 0.2819	*p* = 0.471	***p* = 0.0385**	*p* = 0.5422
18RS HEPE	r = −0.165	r = −0.088	r = −0.107	r = −0.014	r = 0.127	r = 0.153
	*p* = 0.3146	*p* = 0.5926	*p* = 0.5177	*p* = 0.9302	*p* = 0.4415	*p* = 0.3539

Bold font indicates statistically significant correlation; 10(S)17(R)-DiHDAs protectin.

**Table 6:** Correlation of anti-inflammatory metabolites with biochemical parameters of patients after the implementation of the diet.

[µg\mL]	TSH	Anti-TG	Anti-TPO	fT3	fT4	CRP
Resolvin E1	r = 0.109	r = −0.136	r = 0.062	r = 0.109	r = −0.2	r = 0.104
	*p* = 0.5099	*p* = 0.4099	*p* = 0.709	*p* = 0.508	*p* = 0.2218	*p* = 0.528
Resolvin D1	r = 0.254	r = −0.052	r = −0.136	r = 0.203	r = −0.256	r = 0.225
	*p* = 0.1187	*p* = 0.7528	*p* = 0.4081	*p* = 0.2149	*p* = 0.1162	*p* = 0.1688
10S17R DiHDHA	r = −0.166	r = −0.08	r = 0.274	r = 0.111	r = 0.022	r = 0.114
	*p* = 0.3113	*p* = 0.6277	*p* = 0.0907	*p* = 0.5019	*p* = 0.8938	*p* = 0.4895
Maresin 1	r = 0.259	r = 0.014	r = 0.007	r = −0.083	r = −0.164	r = 0.016
	*p* = 0.1107	*p* = 0.9309	*p* = 0.9675	*p* = 0.6157	*p* = 0.317	*p* = 0.9244
17RS HDHA	r = −0.028	r = −0.1	r = 0.156	r = −0.08	r = −0.019	r = 0.184
	*p* = 0.8646	*p* = 0.5447	*p* = 0.3436	*p* = 0.6277	*p* = 0.5715	*p* = 0.2614
18RS HEPE	r = −0.007	r = 0.023	r = −0.017	r = −0.325	r = −0.093	r = −0.214
	*p* = 0.9668	*p* = 0.8903	*p* = 0.9177	***p* = 0.0434**	*p* = 0.9064	*p* = 0.1916

Bold font indicates statistically significant correlation; 10(S)17(R)-DiHDAs protectin.

**Table 7:** Anthropometric characteristics of the study group.

Anthropometric Parameters	Mean ± SD
Age at the beginning of this study [years]	37.393 ± 8.097
Height [cm]	166.729 ± 5.133
Body mass [kg]	71.677 ± 12.293
BMI [kg/m^2^]	25.69 ± 3.883
Body fat mass [g]	24,766.80 ± 8408.336
Body fat percentage [%]	34.857 ± 6.480

BMI—body mass index.

## Data Availability

The data presented in this study are available on request from the corresponding author.
